# Echocardiographic and clinical outcomes following beating heart NeoChord DS1000 mitral valve repair: a single centre case series

**DOI:** 10.3389/fcvm.2023.1160979

**Published:** 2023-06-23

**Authors:** Amy Brown, Hallie L. Jefferson, Ali Fatehi Hassanabad, Christopher Noss, Nicole Webb, Paul W. M. Fedak, William D. T. Kent, Corey Adams

**Affiliations:** ^1^ Department of Cardiac Sciences, Libin Cardiovascular Institute, University of Calgary, Calgary, AB, Canada; ^2^ Department of Anesthesiology, Libin Cardiovascular Institute, University of Calgary, Calgary, AB, Canada

**Keywords:** mitral valve repair, minimally invasive surgery, NeoChord, degenerative mitral regurgitation, artificial neochords

## Abstract

**Background:**

The NeoChord DS1000 system implants artificial neochords transapically, through a left mini-thoracotomy to treat degenerative mitral valve regurgitation (MR). Performed without cardiopulmonary bypass, neochord implantation and length adjustment is guided by transesophageal echocardiography. We describe imaging and clinical outcomes for a single center case series using this innovative device platform.

**Methods:**

In this prospective series, all study patients had degenerative MR and were considered for conventional mitral valve surgery. Moderate to high-risk candidates were screened for NeoChord DS1000 eligibility based on echocardiographic criteria. Study criteria included isolated posterior leaflet prolapse, leaflet-to-annulus index greater than 1.2, and coaptation length index greater than 5 mm. Patients with bileaflet prolapse, mitral annular calcification, and ischemic MR were excluded from our early experience.

**Results:**

Ten patients underwent the procedure, including 6 males and 4 females, with a mean age of 76 ± 9.5 years. All patients had severe chronic MR and normal left ventricular function. One patient required conversion to an open procedure for failure to deploy neochords with the device transapically. The median number of NeoChord sets was 3 (IQR 2.3–3.8). Immediate post-procedure (POD#0) degree of MR on echocardiography ranged from mild or less, and on postoperative day 1 (POD#1) from moderate or less. Average length of coaptation was 0.85 ± 0.21 cm and average depth of coaptation was 0.72 ± 0.15 cm. At 1-month follow-up echocardiography, MR was graded from trivial to moderate and left ventricular inner diameter dimensions decreased from an average of 5.4 ± 0.4 cm to 4.6 ± 0.3 cm. None of the patients who had successful NeoChord implantation required blood products. There was 1 perioperative stroke with no residual deficits. There were no device-related complications or serious adverse events. The median length of hospital stay was 3 (IQR 2.3–10) days. 30-day and 6-weeks postoperative mortality and readmission rates were 0%.

**Conclusion:**

We report the first Canadian case series using the NeoChord DS1000 system for off-pump, transapical, beating heart mitral valve repair, through a left mini-thoracotomy. The early surgical outcomes suggest this approach is feasible, safe, and effective in reducing MR. This novel procedure has the advantage of offering a minimally invasive, off-pump option for select patients with high surgical risk.

## 1. Introduction

Mitral valve repair is the gold standard treatment for patients with chronic degenerative MR (1). With both leaflet resection and preservation repair techniques, there is proven efficacy and long-term durability (1). These conventional surgical techniques can be performed through minimally invasive access, but require cardiopulmonary bypass, with its associated risks. Alternatively, transcatheter edge-to-edge mitral valve repair avoids cardiopulmonary bypass, but with favourable results only in select patient populations (2, 3). For this reason, surgical repair remains the principle strategy for most patients (1–4). The NeoChord DS1000 system (NeoChord, Inc., Minneapolis, MN, USA) has emerged as a hybrid of surgical and transcatheter techniques for mitral valve repair in appropriate patients (4).

The NeoChord DS1000 system is an innovative device that implants GORE-TEX neochords transapically, through a left mini-thoracotomy, to treat MR without the need for cardiopulmonary bypass (“off-pump”). NeoChord implantation and chordal length adjustment is performed under 2D and 3D transesophageal echocardiographic (TEE) guidance under physiologic loading conditions with the heart beating. This provides accurate adjustment of chordal length, achieving a normally functioning mitral valve, and left ventricular (LV) reverse remodelling (4). Although it has proven safety and efficacy, NeoChord DS1000 has had a slow adoption in Canada (4, 5). Here we present the early surgical outcomes for the first Canadian single-centre series of patients treated with the NeoChord DS1000 mitral valve repair system. We also discuss how to overcome challenges in health technology implementation to advocate for continued assessments of new healthcare technology including the NeoChord DS1000 system.

## 2. Materials and methods

In this prospective series, all patients had indications for surgical mitral valve repair due to severe degenerative MR (Carpentier Type II). Considered higher risk for conventional mitral valve surgery, these patients were offered the NeoChord procedure as an alternative approach. Ten patients who had anatomically appropriate valve morphology consented to the procedure.

### 2.1. Echocardiographic eligibility

Patients were screened for NeoChord DS1000 eligibility based on echocardiographic criteria, performed using 3mensio Structural Heart Software version 10.3 (Pie Medical Imaging) by NeoChord. These criteria included isolated posterior leaflet prolapse, leaflet to annulus index greater than 1.2, and coaptation length index of at least 3. The leaflet to annulus index, calculated as the ratio between the sum of the anterior and posterior leaflet height and the anteroposterior diameter, predicts the amount of tissue that could generate the new leaflet coaptation surface post-repair. A value greater than 1.2 has been significantly related to mild MR or less at 1-year follow-up (6). The coaptation length index predicts sufficient overlap of tissue to obtain a postoperative coaptation length of 3–5 mm (7). Anatomical classification of valve morphology was performed as previously described: type A, isolated central posterior leaflet prolapse/flail; type B, posterior multisegment prolapse/flail; type C, anterior or bileaflet prolapse/flail; type D, paracommissural prolapse/flail or any type of disease with significant leaflet or annular calcification (8). Patients with type C and type D mitral valve morphologies were deemed ineligible for this early experience series and not offered this intervention. Anatomical measurements for eligibility were performed using 3mensio Structural Heart Software version 10.3 (Pie Medical Imaging) by NeoChord.

### 2.2. Operative technique

All 10 procedures were performed at our centre after the operative team had travelled for observation and simulation training at an experienced centre. The cases were performed under the guidance of an experienced surgeon proctor. General anesthetic and full hemodynamic monitoring was used in a hybrid operative room. All cases were done off-pump with a perfusionist on standby. TEE images in 2D and 3D were displayed on large monitors, visible to the entire surgical team. An intraoperative cell salvage device was used for transfusion of harvested blood loss.

Patients were positioned supine and, for safety, wires were percutaneously placed in the right common femoral artery and femoral vein using ultrasound guidance. Transthoracic ultrasound was used to identify the left ventricular (LV) apex at the 4th or 5th intercoastal space in the left axillary line. A 3–4 cm incision was made and soft tissues and ribs were retracted. TEE was used to guide appropriate LV apical access, as well as to confirm wire insertion and positioning from the LV apex across the mitral valve. Hemostasis at the LV apex was achieved with two 2-0 Prolene pledgetted sutures, which were placed as a mattress and then snared. Heparin was given to achieve an activated clotting time of greater than 300 s. The NeoChord DS1000 system was inserted through the LV apex and positioned across the mitral valve into the left atrium. The prolapsing or flail P2 segment was grasped, and adequate tissue in the grasper was confirmed using fiber optic feedback, with 4 lights, contained within the NeoChord DS1000 system. The hooked needle of the device was deployed through the leaflet segment. Two ends of the inserted neochord were pulled out through the LV apex. The process was repeated for additional chords, according to TEE evaluation, depending on the degree of residual leaflet prolapse and MR. The chords were passed through a single large pledget, and then with TEE guidance, the chords were tensioned and tied when valve function was optimal. Heparin was reversed using Protamine sulfate. One pleuro-pericardial chest tube was inserted, and the thoracotomy was closed. All patients were transferred to the Cardiovascular Intensive Care Unit for recovery and monitoring.

## 3. Results

Ten patients underwent the NeoChord procedure, 6 males and 4 females with an average age of 76 ± 9.5 years. All patients had significant comorbidities with baseline demographics shown in Table 1. Two patients had open mitral annuloplasties performed years prior. Pre-operative European System for Cardiac Operative Risk Evaluation (EuroSCORE) II values ranged from 0.6% to 3.1%, which numerically reflects relative low operative risks, however not captured in the EuroSCORE were comorbidities including Child-Pugh A Liver Disease, frailty, arthritis and seizure disorder. Pre-operatively, all patients had normal LV function with severe MR. Mean preoperative mitral annulus diameter was 38.6 ± 5.0 mm. Mean leaflet-to-annulus index was 1.3 ± 0.3 and coaptation length index was 4.3 ± 2.7.

**Table 1:** Baseline characteristics and pertinent echocardiographic measurements.

Variable	*N* (%) or *x* ± SD
Age	75.9 ± 9.5
Male sex	6 (60)
EuroSCORE II (%)	1.8 ± 0.9
Cardiovascular risk factors	
Hypertension	3 (30)
Dyslipidemia	5 (50)
Diabetes	0 (0)
Renal disease	0 (0)
Coronary artery disease	5 (50)
Atrial fibrillation	2 (20)
Peripheral vascular disease	1 (10)
Stroke	0 (0)
Prior cardiac surgery	2 (20)
NYHA	
Class 1	3 (30)
Class 2	3 (30)
Class 3	4 (40)
Class 4	0 (0)
Right ventricular systolic pressure (mmHg)	
<25	1 (10)
25–40	3 (30)
41–55	2 (20)
>55	4 (40)
Left ventricular ejection fraction	
<30%	0 (0)
31–54	0 (0)
>54	10 (100)
Tricuspid regurgitation	
None/trivial	4 (40)
Mild	4 (40)
Moderate	2 (20)
Severe	0 (0)
Mitral annulus diameter (mm)	38.6 ± 5.0
MV leaflet-to-annulus index	1.3 ± 0.3
Coaptation length index	4.3 ± 2.7

**Figure 1:**
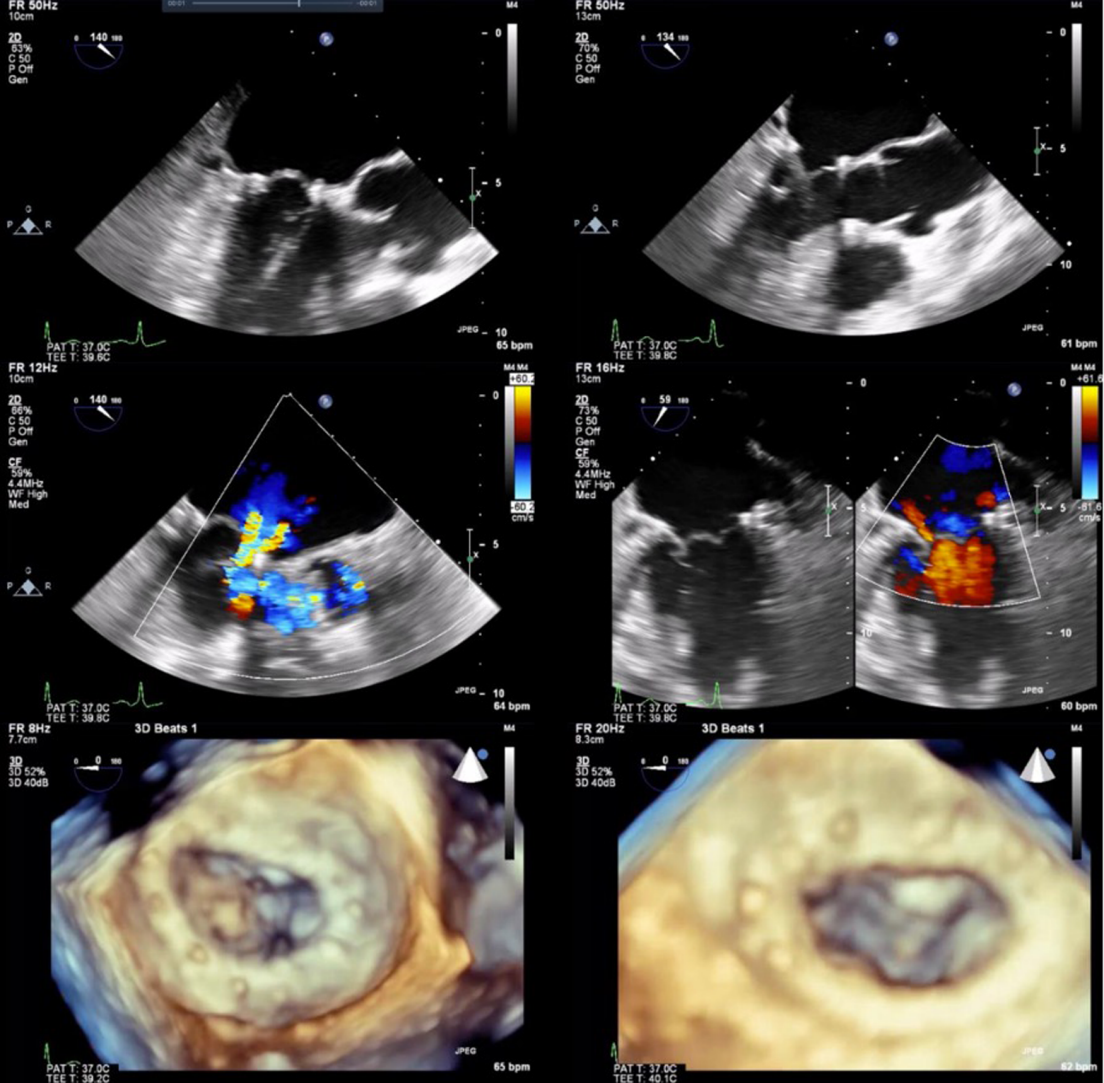
Two and 3-dimensional view of preoperative images (**left** column) with P2 prolapse and severe MR. Post intraoperative delivery of NeoChord system and NeoChord tightening with mild residual MR (**right** column).

The NeoChord DS1000 was successfully implanted in 9 out of 10 patients transapically, with representative images in Figure 1. One patient required conversion to an open procedure for failure of the device to adequately grasp and deploy the chords. After sternotomy and cardiotomy with visual inspection of the mitral valve, the leaflet tissue was fragile and consistent with fibroelastic deficiency, with torn tissue from the device grasper. The patient required mitral valve replacement and was removed from further analysis of the NeoChord DS1000 procedural results.

The median number of NeoChord sets was 3 (IQR 2.3–3.8) (Table 2). Perioperative outcomes as defined by the Mitral Valve Academic Research Consortium are shown in Table 2. None of the patients who had successful NeoChord implantation required blood products. There were no bleeding complications. One patient experienced a perioperative embolic stroke with no residual deficits. Eight of the 9 patients were extubated in the operating room or within 6 h of the procedure. The median length of intensive care stay was 1 (IQR 1-1) days and median total hospital length of stay was 3 (IQR 2.3–10) days. The fourth patient in the series had significantly longer ICU and total length of stay due to ventilator-dependence and slow convalescence related to pre-existing aphasic dementia. Hemodynamically stable bradycardia was experienced by 4 patients with spontaneous recovery, and no patient developed an indication for permanent pacemaker insertion. Overall, 30-day and 6-week postoperative mortality was 0%. Additionally, 6-week hospital readmission rates were 0%.

**Table 2:** Periprocedural outcomes.

Outcome	*N* (%) or Median (I–III Quartile)
Implanted Neochords	3 (2.3–3.8)
Intraoperative complications	
Conversion to conventional surgery	1 (10)
ECMO	0 (0)
IABP	0 (0)
Major or extensive bleeding	0 (0)
Mechanical ventilation time	
0 h	5 (55.6)
0–6 h	3 (33.3)
>6 h	1 (11.1)
Reintubation	1 (11.1)
ICU length of stay (days)	1 (1–1)
Hospital length of stay (days)	3 (2.3–10)
Periprocedural complications	
Stroke	1 (11.1)
TIA	0 (0)
Myocardial infarction	0 (0)
Acute kidney injury	0 (0)
Rhythm disturbances	4 (44.4)
Permanent PM insertion	0 (0)

**Figure 2:**
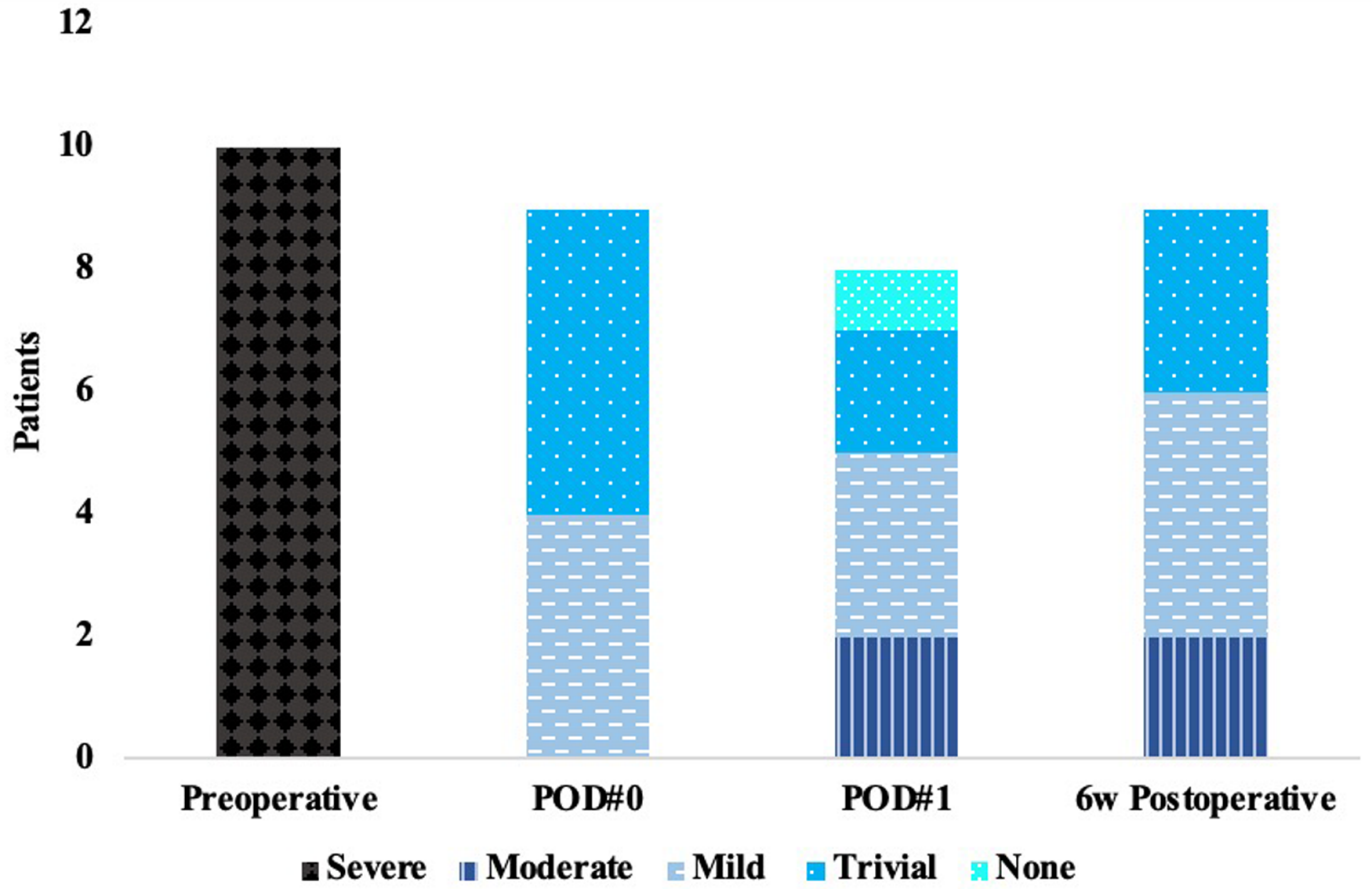
Echocardiographic measurements of mitral regurgitation.

Qualitative intraoperative post repair TEE found trivial MR in 5 patients and mild MR in 4 patients. Average length of coaptation was 0.86 ± 0.20 cm and average depth of coaptation was 0.72 ± 0.15 cm. On POD#1, there was no MR in 1 patient, trivial in 2 patients, mild in 3 patients, and moderate in 2 patients (Figure 2). One patient did not have a transthoracic (TTE) on POD#1. At 6-week TTE follow-up, the degree of MR ranged from trivial in 3 patients, mild in 4 patients, and moderate in 2 patients. Left ventricular inner diameter in diastole (LVEDD) decreased from an average of 5.4 ± 0.4 cm preoperatively to 4.6 ± 0.3 cm, measured 6 weeks postoperatively, a 15% decrease. Mean left ventricular ejection fraction (LVEF) preoperatively was 61%, 54% on POD#1, and was 52% at 6 weeks postoperatively.

## 4. Discussion

This single centre case series describes the first reported Canadian experience with the NeoChord DS1000 system for the treatment of degenerative MR. Using echocardiographic predictors of successful coaptation index, selected patients were offered this procedure. The short term clinical and echocardiogram results are favourable with mild MR or less in 7 patients and moderate MR in 2, at 6 weeks postoperatively. One patient required conversion to open surgical intervention, highlighting the importance of stringent patient selection. Other reports of treatment success with low procedural and 30-day complications validate the early outcomes demonstrated here (4, 8, 9).

The NeoChord DS1000 system is a relatively novel technique for the treatment of degenerative MR, with the first successful outcomes reported in 2014 (10). This study demonstrated both efficacy, with 85% of patients (*N* = 13) having mild MR at 6 months postoperatively, and safety, with no procedural complications (10). Subsequently, while over 1,200 cases have been performed worldwide, limited reports on midterm results are available (11). Kiefer and colleagues reported 5-year clinical and echocardiographic results on a small case series (9). They described persistent improvement in cardiac symptoms, no recurrence of severe MR, and a trend toward left ventricular remodelling (9). Furthermore, D'Onofrio and colleagues reported 5-year clinical and echocardiographic outcomes of 100 patients (11). A 23.7% cumulative incidence of severe MR and 16.7% reoperation rate was found with NYHA I or II symptoms at 5-years (11).

Our experience is similar to previous series, demonstrating a 10% rate of conversion to open surgery. Reports of procedural failure range from 0% to 20%, with the highest rates in the first 20 procedures performed (4, 5, 8, 12). An examination of the learning curve suggests that completion of 49 cases is required to develop a failure rate of 5% or less (12). Therefore, our rate of conversion is within the expected range for early adoption of this novel technology. Hand-eye coordination, with real time instrument guided object grasping and manipulation, coordinated with 2D and 3D echocardiographic images requires time to develop. Technical issues related to procedural failure, include entangling of the anterior mitral leaflet subvalvular apparatus, leaflet injury, and chordal over-tensioning, can result in chordal rupture (12). In addition to technical factors, our experience suggests that patient factors such as fragile leaflet tissue and frailty should be considered higher risk for perioperative and postoperative complications.

By not including a ring annuloplasty, the NeoChord DS1000 system challenges some traditional principles of mitral valve repair (9, 13). The lack of annular support has the potential to impact the longevity of NeoChord DS1000 system repair, but as yet, there is no supportive evidence. A propensity-matched study comparing outcomes of conventional mitral valve repair using annuloplasty ± neochord implantation to the NeoChord procedure, demonstrated significantly less days spent in ICU, less intubation time, less bleeding complications, and less new-onset atrial fibrillation in the NeoChord cohort (14). Numerically more patients in the NeoChord cohort had moderate or severe MR postoperatively, and this translated to less freedom from moderate MR at 5-year follow-up. However, in patients with Type A anatomy, no difference in recurrent severe MR of 79% was found (14). This suggests that perioperative morbidity is improved following the NeoChord procedure, with no compromise in clinical outcomes compared to surgical mitral valve repair in select patients. A Randomized Trial comparing the Neochord DS1000 System to Open Surgical Repair [(Rechord); ClinicalTrials.gov Identifier NCT02803957] is currently in progress.

Similar to previous reports, our series found a decrease in LV end diastolic diameter, supportive of reverse remodelling (9). Early correction of severe MR before extensive LV remodelling occurs may alter the disease process and prevent LV dilation and annular enlargement. It is likely that patients with the greatest potential for a favourable long-term outcome with NeoChord are those with posterior leaflet prolapse and no annular dilatation as shown in our series, or redo cases with an annuloplasty ring *in situ*. A study of 100 patients who underwent the NeoChord procedure demonstrated that patients with isolated posterior leaflet prolapse/flail had significantly less recurrence of severe MR at 5 years compared to patients with anterior or bileaflet prolapse/flail, paracommissural prolapse/flail or any presence of leaflet/annular calcification; 14.7% vs. 63%, respectively (11). Additionally, a multicentre study described outcomes of patients with previous mitral valve repair who underwent the NeoChord procedure (15). All patients had had an annuloplasty. Technical success was achieved in 100% of 15 patients with no in-hospital deaths or major complications. At 2-years follow-up, 50% of patients had none or trivial MR, 3.3% had mild MR, and 16.7% had moderate MR. Eighty-three percent of patients had NYHA I symptoms (15). This report suggests that reintervention with the NeoChord procedure may offer less morbidity and mortality than redo surgical intervention with similar clinical results.

Our early clinical and echocardiographic outcomes using the NeoChord DS1000 system are aligned with current consensus of previously published reports. This contribution is important as less invasive procedural options for mitral valve disease is a growing field with rapid advances in healthcare devices (4). Careful study of mitral valve repair device technologies should be balanced with the evolving demand for minimally invasive options. This requires outcomes assessment, ideally through randomized controlled trials comparing safety and efficacy of the device compared to accepted standard of care. This is arguably a weak area in the assessment of the NeoChord DS1000 system, with few clinical trials completed. This may be a reason for the slow adoption of this technology in Canada. Furthermore, once approved by regulatory bodies, new device implementation requires buy-in from multistakeholders for implementation in the operating room (16).

The adoption of any new medical device can disrupt work routines, even in a highly structured environment like the operating room (17). Successful implementation requires adjustments on an organizational and interpersonal level to allow for a collective learning process and development of new practices (17). Cardiac surgery, in particular, follows standardized routines that usually differ only in some details of surgical technique and operation room layout. Cardiac surgery thrives on consistency, which can slow the adoption of new procedures and devices (17). Institutional factors, systemic policies, patient culture and values, funding, and physician preference have been identified as barriers for the uptake of innovation in cardiac surgery (18). Saka and Ferenbok (2021) published recommendations for implanting change in the clinical environment including early involvement of stakeholders, practical team training, early involvement of patient and physician perspectives, involvement of champions, and adoption of a collaborative funding model to secure ongoing funds and reduce cost (18). A thoughtful consideration of these strategies is vital for any organization attempting to introduce innovative devices, such as the NeoChord DS1000 system and will likely improve uptake.

Our early surgical outcomes using the NeoChord DS1000 system for off-pump, transapical, beating heart mitral valve repair through a left mini-thoracotomy suggest this approach is feasible for adoption into an experienced mitral valve repair program and both safe and effective for selected high-risk patients. It can be considered an alternative, minimally invasive strategy for higher risk patients with degenerative MR that have posterior leaflet prolapse or flail segments, with limited annular dilation. While the safety and feasibility of this technology has been previously reported, our results demonstrate novelty in the Canadian healthcare system. Furthermore, we support the introduction of this technology into other Canadian centres, and the introduction of NeoChord DS1000 into the US.

## Data Availability

The raw data supporting the conclusions of this article will be made available by the authors, without undue reservation.
